# The postpartum return-to-home transition: A narrative review

**DOI:** 10.18332/ejm/241927

**Published:** 2026-10-31

**Authors:** Mónica Alexandra Pinho da Silva, Marcia Regina Cubas, Alexandrina Maria Ramos Cardoso

**Affiliations:** 1 Instituto de Ciencias Biomedicas Abel Salazar, Universidade do Porto, Porto, Portugal; 2 Escola Superior de Enfermagem, Universidade do Porto, Porto, Portugal; 3 Pontificia Universidade Catolica do Parana, Curitiba, Brazil; 4 RISE-Health, Escola Superior de Enfermagem, Universidade do Porto, Porto, Portugal

**Keywords:** postpartum period, postnatal care, hospital to home transition, nurse-midwives, transitions theory

## Abstract

Shortened postpartum hospital stays worldwide have intensified the need for effective discharge preparation and continuity of care. The postpartum return-to-home transition remains underexplored within nursing-midwifery scholarship, limiting the development of structured approaches to support women and families during the early postpartum period. Using Meleis’ Transitions Theory as an analytic lens, a narrative review was conducted to examine the nature of this transition, its conditions, patterns of response, and nursing-midwifery therapeutics. Searches were carried out in PubMed, CINAHL and Scopus (March 2026) and seven sources (five empirical studies and two non-empirical articles) met the inclusion criteria, three of which concerned the neonatal intensive care context; findings were extracted and mapped deductively onto the components of the theory. Transition conditions identified included parity, mode of birth, infant feeding, and parental knowledge. Discharge readiness was the most frequently measured pattern of response. Only one study assessed outcomes after discharge: lower readiness predicted post-discharge coping difficulty. Discharge education was the most frequently examined nursing-midwifery therapeutic. Structured continuity-of-care interventions, including home visits and telehealth, were also identified as important but inconsistently provided. The nature of the transition is conceptualized as a nested situational transition within the developmental transition to parenthood, refining Transitions Theory for nurse-midwives’ practice in the postpartum period. The proposed framework may help inform nurse-midwives’ assessment of transition conditions at discharge and the planning of follow-up, by addressing the full range of adaptive demands women and families face at home. The model requires empirical validation, particularly in routine postpartum care.

## INTRODUCTION

Over recent decades, the average length of hospital stay after childbirth has shortened, driven by organizational reforms, financial pressures, and advances in obstetric and neonatal care. Early discharge (variably defined from 12 hours to 72 hours depending on the country) does not consistently worsen key clinical outcomes when accompanied by adequate discharge support^1,2^. However, parents’ experiences of the period preceding infant hospital admission within the first four weeks after birth, highlight the challenges families may face at home^3^.

Parents’ experiences of early discharge highlight the importance of adequate information and support during the transition home^4^. Higher quality discharge teaching predicts greater discharge readiness^5,6^, which in turn predicts fewer coping difficulties and lower unplanned readmission rates during the early postpartum weeks^6^. Parents frequently report feeling underprepared for infant care, breastfeeding challenges, and self-care in the early weeks^3,7,8^. Systematic assessment of parental competencies has confirmed significant gaps documented in areas such as breastfeeding, health promotion, and recognition of warning signs^9-11^.

The quality and timing of discharge planning vary across settings. Stakeholders (women, partners, families, nurse-midwives) describe the discharge process as rushed, variable in content, and insufficiently responsive to social and emotional needs, particularly where time and staffing constraints are present^12-14^. Nursing documentation and clinical attention remain disproportionately oriented toward biomedical and functional diagnosis, with concepts related to parental role, maternal knowledge, and family adaptation receiving comparatively less attention^15^.

Transitions Theory by A. Meleis defines transitions as complex processes that occur when individuals move from one life phase, condition, or environment to another^16^. They are temporally unfolding, multidimensional processes characterized by changes in identities, roles, relationships, and behaviors, shaped by facilitating and inhibiting conditions across personal, social, community, environmental, and system levels^17^. Transitions Theory identifies several key components relevant to understanding transitional experiences: the nature of the transition, the conditions that facilitate or inhibit the transition, patterns of response and nursing-midwifery therapeutics^17^. The nature of transitions refers to characteristics such as the type of transition, the degree of change involved, and the duration of the process. Transition conditions include personal, social, and environmental factors that may facilitate or inhibit adaptation. Patterns of response reflect the ways in which individuals and families respond to transitions, including indicators of healthy adaptation or vulnerability. Nursing-midwifery therapeutics refer to intentional interventions designed to support individuals and families during transitions^18^.

The transition to parenthood represents a particularly complex developmental transition, involving the reorganization of individual and family roles, relationships, goals, and responsibilities^19^. Nursing and midwifery care based on Transitions Theory has been associated with enhanced maternal role satisfaction, increased parental self-efficacy, and improved maternal adaptation^20^.

Combining Transitions Theory with complex concepts encourages a more holistic understanding of transition processes and highlights the potential to improve outcomes through theory-informed practice^21^. In the case of the postpartum return-to-home process, research has largely focused on discharge readiness^13,14^ or parental experiences in isolation^7^, while the transition itself has rarely been examined. Applying Transitions Theory to the postpartum return-to-home process may help generate a more practice-relevant understanding of this transition. This review aims to examine the nature of the transition, the conditions that shape it, patterns of response, and the interventions from nurse-midwives that can support it.

A narrative review methodology was chosen since narrative reviews are suited to synthesizing a body of literature to build conceptual understanding, offering a more integrative and interpretive account of a research area^22^. Eligible studies were required to address the postpartum hospital-to-home transition or discharge preparation in the early postpartum period, defined as the period from birth to six weeks after birth, and to apply Transitions Theory as an explicit conceptual or guiding framework. Both conditions were necessary for inclusion, as the aim of the review was not to survey the discharge readiness literature broadly but to examine how nursing theory has been applied to this specific transition. Studies addressing discharge preparation for parents of preterm infants were also considered when grounded in Transitions Theory, as they provide relevant insights into discharge readiness and parental adaptation during the postpartum hospital-to-home transition.

Eligible designs comprised empirical studies of any design (quantitative, qualitative, or mixed methods) and non-empirical sources (reviews and theoretical or conceptual papers), since the aim of the review was conceptual. The population comprised mothers, fathers, or other parents of term or preterm infants, with no restriction by maternal age; studies that also included healthcare professionals were eligible when they addressed parents’ discharge preparation. Eligible settings were hospital maternity wards and neonatal units, with the transition followed into the home. Sources were excluded when Transitions Theory was not used as a conceptual or guiding framework or when the hospital-to-home transition was not a primary focus.

Key search terms used were: postpartum, postnatal, hospital discharge, discharge readiness, hospital-to-home transition, Transitions Theory, Meleis, nursing theory; they were combined with the Boolean operators AND and OR. No restrictions were applied regarding publication date or language. The search was conducted on 30 March 2026 across three electronic databases: PubMed (n=11), Scopus (n=5) and CINAHL (n=5), yielding a total of 21 records. After removing three duplicates, 18 records were screened by two authors, and six records were excluded at the title and abstract level. Full-text review was conducted by two authors, with a third author resolving disagreements. Of the 12 full-text articles reviewed, five were excluded: two did not apply Transitions Theory as a conceptual or guiding framework; in the other three, the postpartum hospital-to-home transition was not a primary focus of the study. Seven sources were included in the final review. A PRISMA 2020 flow diagram^23^ is provided in the Supplementary file.

For each source, the following information was recorded: authors, year and country; design and methods; setting and population (routine postpartum care or neonatal intensive care); how Transitions Theory was used; the type(s) of transition described; transition conditions; patterns of response, including how and when they were measured; nursing-midwifery therapeutics, including their timing (antenatal, during hospitalization or after discharge) and the professionals delivering them; and main findings. Data were extracted by one author and verified against the original sources by a second author; disagreements were resolved through discussion and, where needed, by a third author.

Analysis followed a deductive approach, using the four components of Transitions Theory (nature of the transition, transition conditions, patterns of response and nursing-midwifery therapeutics). Extracted findings were organized in a synthesis table in which each finding from each source was assigned to one of these components. The mapping was performed by one author and reviewed by a second author. Contradictory findings were not reconciled but reported side by side, with attention to the design and setting of each source, and information that did not fit the framework, such as findings specific to neonatal intensive care, was retained and identified as context-specific.

The definition of a nested transition was derived interpretively in three steps: first, we compared how each source characterized the type, sequence, and boundaries of the transitions it described, including the multiple and hierarchically related transitions identified in the two non-empirical neonatal sources; second, we contrasted these accounts with the treatment of multiple transitions in Transitions Theory^16,17^; and third, we abstracted the features shared by the return-home transition into four defining criteria, which were then checked against the sources on term infants in routine postpartum care.

The seven studies included in this review were conducted across five countries: United States, Denmark, Turkey, China and Canada. All seven sources were published between 2009 and 2026. The five empirical studies employed diverse methodological approaches: two quantitative correlational designs^6,24^, one randomized controlled trial^25^ and two qualitative studies, namely an ethnographic field study^26^ and a qualitative descriptive study^27^. The two non-empirical sources consist of a narrative review^28^ and a conceptual paper^29^. Key characteristics of all included sources are provided in Supplementary file Table S1. Consistent with the interpretive and conceptual aim of narrative reviews^22^, no formal critical appraisal of methodological quality was undertaken.

The sources contribute different types of evidence to the synthesis, and their findings are interpreted accordingly. The two correlational studies, both conducted in routine postpartum care, examine associations between transition conditions, the quality of discharge teaching delivered by nurses and discharge readiness, but cannot establish causality; only one of them followed mothers after discharge, at three weeks^6,24^. The randomized controlled trial is the only source testing an intervention: an online infant-care education program delivered antenatally, during the last trimester of pregnancy^25^. The two qualitative studies illuminate how parents and professionals experience discharge preparation and the first days at home^26,27^. The two non-empirical sources, a narrative review^28^ and a conceptual paper^29^, both focused on neonatal intensive care, contribute mainly to the theoretical conceptualization of the nature of the transition rather than to empirical evidence. Three of the seven sources therefore address neonatal rather than routine postpartum discharge^27-29^. Regarding the professionals involved, the sources examined care provided by nurses, midwives and multidisciplinary maternity and neonatal teams, and the professional qualification of those providing care was not always specified; findings are therefore attributed to nursing and midwifery care in general.

## DEVELOPMENTS

### Conditions shaping the transition

Transition conditions were the most extensively represented component, appearing in all seven sources, operating at multiple levels, including individual, interpersonal and health system contexts. These conditions may either facilitate or inhibit parents’ adaptation following discharge from hospital. At the personal level, the most consistently identified conditions were parity^6,27,29^, mode of birth^24,25^, infant feeding method^6,24,25^, parental knowledge and confidence at the start of the transition^25-27^, maternal age^29^, and socioeconomic conditions^27^. In this review, the knowledge and competence that parents bring into the transition (initial competence) are treated as conditions, whereas the competence acquired through the transition is treated as a pattern of response.

First-time mothers report greater uncertainty and reliance on professional guidance, whereas multiparous women may draw on their previous experiences to navigate the early postpartum period more confidently^24,26^. The mode of birth can influence maternal recovery and the level of support required during the first days at home, with women who experience cesarean births often requiring additional guidance and instrumental support regarding physical recovery and infant care^24,25^. Breastfeeding women, when compared with exclusively bottle-feeding mothers, seem to experience additional learning demands and require sustained support from nurses and midwives^24,25^. Higher maternal knowledge of infant and self-care before discharge predicted greater discharge readiness and better early coping^6^. In a conceptual analysis, adolescent mothers were described as having lower confidence in newborn care^29^, and, in a neonatal context, parents with lower socioeconomic resources and lower health literacy may face additional challenges, including limited access to social support, financial constraints and reduced availability of community services^27^. Beyond the included sources, these findings resonate with current international guidance emphasizing that postnatal care must be individualized, responsive to women’s circumstances and designed to identify those requiring additional support after discharge^30,31^.

At the interpersonal level, partner and family involvement emerged as a facilitating condition^26-28^. Father co-admission and inclusion in professional guidance were described as central to first-time parents’ development of a shared parental identity^26^. Social support from partners, extended family and community networks was consistently described as an important facilitator of adaptation, with parents who anticipated practical and emotional support at home reporting greater readiness for discharge^27^.

Health system conditions appeared in all sources. Studies highlighted variability in discharge preparation practices, including differences in the timing, content, and comprehensiveness of discharge teaching^26,27^. Time constraints and staffing pressures were also described as factors influencing the depth and quality of discharge preparation^26^. Structural barriers, such as restrictive visitation policies^27^, the absence of systematic antenatal education provision^25^, and poor hospital-to-community coordination^26,28^, were identified as inhibitors. Community-level resources, including the lack of primary care availability and community health services, were identified as critical inhibiting conditions^27,28^.

### Patterns of response to the transition

Patterns of response can encompass process indicators, which signal whether the transition is moving towards health or towards vulnerability, and outcome indicators, which reflect its completion^16^. Discharge readiness was the most frequently measured pattern of response^6,24,25^ and is best understood as a process indicator: it captures parents’ perceived preparedness at a single point in time, immediately before discharge, and does not, on its own, demonstrate sustained adaptation after the return home. It reflects a complex and multidimensional construct encompassing physical recovery, knowledge, coping ability, and anticipated social support^6,24,25^. Beyond the included sources, this is coherent with broader international literature showing that discharge preparation and discharge readiness are multidimensional constructs involving the need for physiological stability, knowledge and confidence in self and infant care, social support, and linkage with follow-up community services^14^. Outside the reviewed corpus, recent evidence also suggests that readiness is not a homogeneous state but may present distinct profiles among women, reinforcing the need for individualized assessment rather than standardized discharge planning^5^.

The qualitative studies and the narrative review described further patterns of response, such as parental self-efficacy, competence and mastery^26-28^. Consistent with Transitions Theory, developing confidence, self-efficacy and coping are read in this review as process indicators, whereas mastery, that is, the competence in infant care and self-care acquired through the transition, together with the integration of the new parental identity, are outcome indicators that can only be assessed after a period at home^16^. In the narrative review, parents of preterm infants were described as concerned about their ability to recognize infant health problems, manage feeding difficulties, and respond to unexpected situations^28^. In the ethnographic study, first-time parents described the early days at home as a learning period during which they refined their caregiving skills and adapted routines to meet the needs of their newborn^26^. Parents of preterm infants described a gradual process of gaining confidence and integrating caregiving practices into everyday life, supported by ongoing communication with healthcare professionals and community services^27^.

Only one study assessed patterns of response after discharge, three weeks after the return home^6^. It established a sequence in which the quality of discharge teaching predicted readiness; lower readiness predicted greater post-discharge coping difficulty; and coping difficulty, together with nurses’ teaching skill, predicted the use of family support and post-discharge health services^6^. These forms of service use are not equivalent: planned help-seeking, such as calling or contacting a health professional, may reflect an appropriate recognition of needs, whereas unplanned emergency visits and readmissions are more likely to signal unmet needs. Rehospitalization was identified as a key adverse population-level outcome^28^. Long-term outcome indicators, such as mastery of infant care and integration of the parental role, were not assessed in any of the included sources.

### Nursing-midwifery therapeutics supporting the transition

Discharge education was the most frequently examined therapeutic. Two forms were described and should be distinguished: education provided during the postpartum hospital stay or immediately before discharge^6,24,26,27^, and antenatal preparation^25^. In the two correlational studies, the quality of in-hospital discharge teaching, in particular the gap between the information mothers needed and the information they received and nurses’ skill in delivering it, explained the largest share of the variance in readiness^6,24^ and, in one study, was indirectly related to post-discharge coping through readiness^6^. Qualitative findings complemented these associations: clear information, opportunities for questions, reinforcement of key infant care skills and individualized, responsive teaching with hands-on learning were valued by parents of preterm infants^27^. A range of topics are prioritized: recognition of warning signs, infant sleep, breastfeeding, introducing the baby to siblings and pain management after cesarean birth^24^. The only intervention study in this review was a randomized controlled trial of online infant care education based on Transitions Theory, delivered during the last trimester of pregnancy; compared with routine care, it improved breastfeeding success (r=0.75) and discharge readiness (r=0.52)^25^. No included study tested the effectiveness of in-hospital discharge education.

Another important therapeutic intervention involved emotional support and reassurance. Parents frequently emphasized the value of supportive interactions with nurses, midwives, and nurse-midwives, particularly during the final days of hospitalization when they were preparing to assume the responsibility of caring for their newborn^26^. These interactions help validate parental concerns and reinforce parenting competence.

Continuity of care between hospital and community services also emerged as a key therapeutic strategy. Structured follow-up contacts, including home visits, telehealth contacts and early postpartum consultations, were described as important interventions for maintaining support after discharge and addressing emerging concerns during the early postpartum weeks^26,28^.

### Conceptualizing the nature of the transition

Most sources conceptualized the transition primarily as situational: a change in environment and context triggered by the event of hospital discharge. The developmental dimension, becoming a parent, was foregrounded only by one source, who addressed both practical skill acquisition and the psychological and identity changes of entering the maternal role^25^, and partially by another, who described the postnatal ward stay as a protected space within which first-time parents took their initial steps toward parenthood^26^. The richest theoretical accounts came from the narrative review^28^, which described the transition as a nonlinear, cyclical process involving at least two simultaneous major transitions (to parenthood and from hospital to home) explicitly invoking Meleis’ taxonomy to argue that multiple transition types coincide in parents of preterm infants, and from the conceptual paper^29^, which identified four intersecting transition types experienced simultaneously by adolescent mothers at NICU discharge: health-illness, developmental (to motherhood), situational (NICU to home), and developmental (adolescent to adulthood), establishing a hierarchical relationship between transition types that no other source in the corpus articulates.

Discharge represented a threshold moment in which responsibility for infant care shifted from healthcare providers to families^26-28^. It can be conceptualized as a multiphase process comprising three interconnected stages: 1) the hospitalization phase, during which discharge preparation should be initiated as early as possible; 2) the discharge, where short-term outcomes can be measured, such as readiness for discharge; and 3) the post-discharge period, when mothers and fathers are confronted with infant care demands at home and their need for support from family and healthcare services^6^.

The findings of this review position the postpartum return-to-home transition as situational in nature for most sources, triggered by the critical event of hospital discharge, while remaining embedded in the ongoing developmental process of becoming a parent. Transitions Theory acknowledges that multiple transitions can coexist and overlap; however, it does not fully theorize the possibility that one transition is contained within, and fundamentally shaped by, another. A hierarchical, nested relationship between transitions was already articulated by Orr et al.^29^ for adolescent mothers at neonatal intensive care discharge, and the nesting concept in this review draws mainly on this source and on the narrative review of parents of preterm infants^28^. What this review adds is threefold: it transfers the idea of nesting to routine postpartum discharge and examines it against the term-birth sources^6,24-26^; it proposes a formal definition with four explicit criteria; and it sets operational boundaries for the nested transition.

A nested transition is a contextually and temporally bounded subprocess embedded within an overarching transition trajectory, characterized by: 1) a discrete precipitating event; 2) a shift in environment or context; 3) intensified role adaptive demands; and 4) distinct patterns of response; while remaining ontologically continuous with the wider transition in which it is contained.

In the context of the transition to parenthood, the return home following childbirth meets all four criteria. It is precipitated by the discrete event of hospital discharge; it involves a fundamental shift from institutional to domestic environment; it concentrates role adaptive demands (caregiving, breastfeeding, physical recovery, interpersonal reorganization) within a narrow temporal window; and it produces measurable patterns of response, including discharge readiness and post-discharge coping difficulty, that are distinct from, and may be related to, long-term parental adaptation. The proposed framework in Figure 1 may offer a theoretically grounded basis for the assessment of transition conditions, identification of families at risk of suboptimal adaptation, and alignment of nursing and midwifery interventions with the specific adaptive demands of this period.

**Figure 1:**
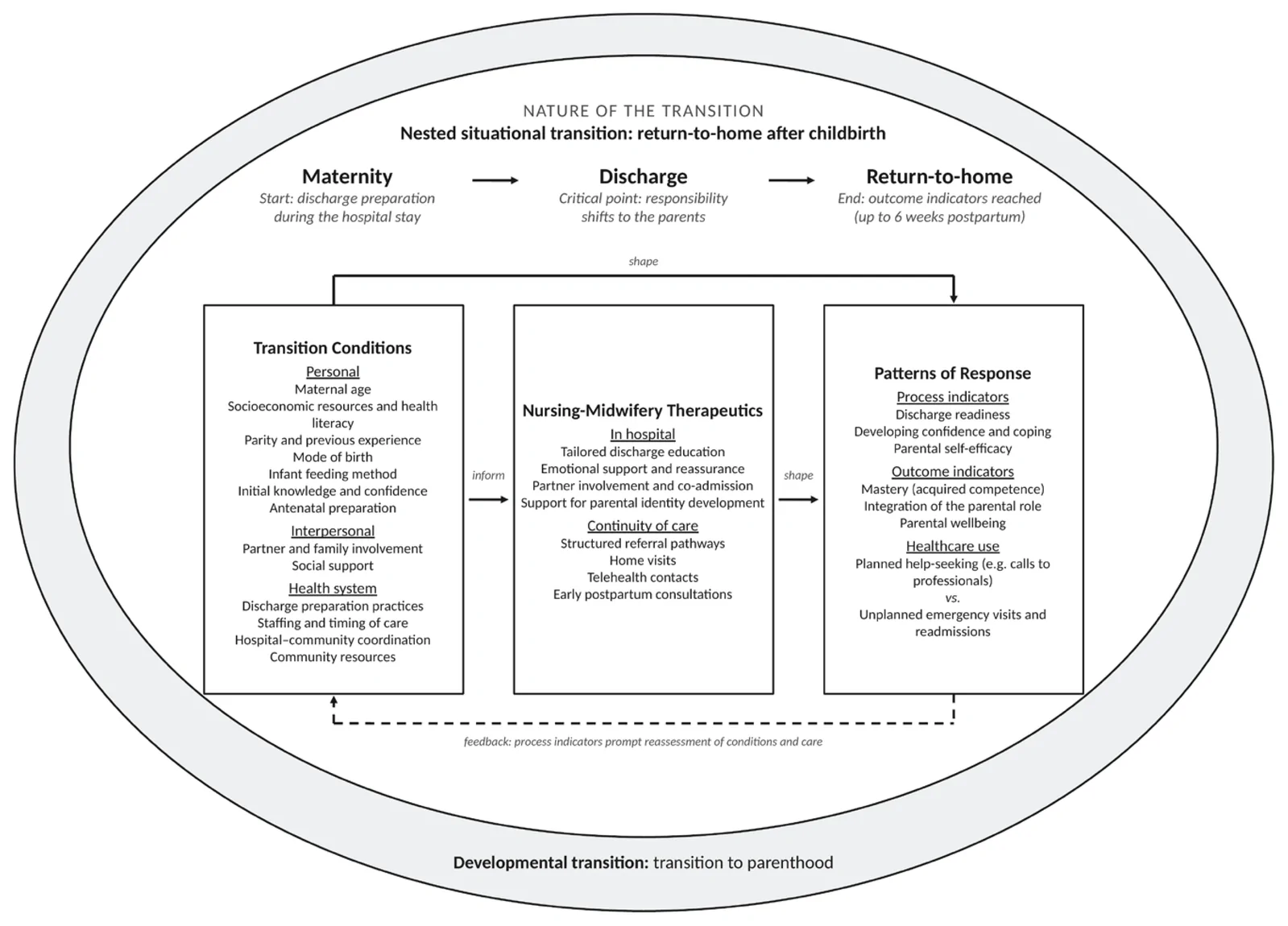
Conceptual framework of the postpartum return-to-home transition as a nested transition within the transition to parenthood, derived from the seven sources included in this review

We propose that the nested transition begins with the initiation of discharge preparation during the postpartum hospital stay, with antenatal preparation acting as an anticipatory condition rather than as part of the transition itself^6,25^. Its critical point is hospital discharge, when responsibility for care shifts from professionals to the family^26-28^. It ends when outcome indicators are reached, that is, when parents demonstrate mastery of infant care and self-care at home, and follow-up has been taken over by community services. For research purposes, this end point may be bounded by the end of the six-week postnatal period^30^, while the developmental transition to parenthood continues beyond it.

### Implications for nursing-midwifery practice, policy and education

The included sources examined care provided by nurses, midwives and multidisciplinary teams; the implications below are framed for nurse-midwives, given their role in postnatal care in many settings, but may apply to all professionals involved in postnatal discharge. Nurse-midwives are centrally positioned to assess transition conditions across individual, interpersonal and system levels and to target interventions accordingly. Tailored discharge education remains important, since the quality of teaching, particularly closing the gap between the information parents need and the information they receive, was the factor most consistently associated with readiness^6,24^. Nurse-midwives’ interventions may also need to support parental identity development, self-efficacy and role confidence to facilitate healthy adaptation across the full developmental transition. Continuity of care interventions, including structured referral pathways, home visits, telehealth and early postpartum consultations by nurse-midwives, may also be important and, outside the reviewed evidence, form part of current international guidelines^30,31^.

Based on the conditions and patterns of response identified, assessment before discharge may include: parity and previous experience; mode of birth and physical recovery; infant feeding method and progress with breastfeeding; parents’ initial knowledge and skills in infant care, self-care and recognition of warning signs; maternal age; socioeconomic resources and health literacy; the support anticipated at home; access to community services; and discharge readiness^6,24-29^.

Referral or enhanced follow-up may be considered when low readiness is identified at discharge, since it predicts later coping difficulty^6^, and when conditions associated with greater adaptive demands accumulate, such as first-time parenthood with low confidence, cesarean birth with limited support at home, unresolved breastfeeding difficulties, adolescent motherhood, limited socioeconomic resources or health literacy, and infants with neonatal care needs^6,24-29^.

The father, partner or another significant person may usefully be involved throughout the hospital stay, through co-admission where possible, inclusion in teaching sessions and in the discharge conversation, and assessment of the support they expect to provide at home, as these were described as facilitators of a shared parental identity and of readiness^26,27^.

These findings also need to be considered in relation to the ongoing policy trend of shorter postpartum hospital stays. Early discharge is not inherently problematic, but without structured preparation and responsive follow-up it may intensify vulnerability at the point of greatest adaptive demand. In settings where early discharge is routine, policy attention may usefully focus on safeguarding quality through adequate midwifery staffing, training, continuity of care and minimum post-discharge contact standards.

Continuity of care requires structured mechanisms that ensure the timely transfer of information between maternity services and community healthcare providers. We suggest the need for structured referral models to support the postpartum return-to-home transition. Such models may facilitate the systematic identification of families requiring additional support, enable coordinated follow-up in primary care services, and strengthen communication between hospital and community-based nurse-midwives.

For education, midwifery curricula may benefit from explicitly addressing postpartum transitional care through a Transitions Theory lens, including discharge planning, parental competency assessment, breastfeeding support and psychosocial vulnerability. Understanding the nested structure of the transition may help nurse-midwives to assess not only whether parents are ready to leave hospital, but whether the conditions for healthy adaptation across the full developmental transition are in place.

### Limitations

Several limitations should be acknowledged. The small number of included studies reflects the limited state of theory-informed research on this transition, and the evidence base was heterogeneous in design and setting, with most studies conducted in high-income countries, limiting transferability. Three of the seven sources were set in neonatal intensive care^27-29^, which differs from routine postpartum discharge and warrants cautious interpretation. Restricting eligibility to sources that explicitly used Transitions Theory was necessary for the conceptual aim but introduces selection bias, as relevant evidence on discharge preparation that did not use this theory, including studies of routine postpartum discharge, was excluded. This inherent theoretical lens bias means the proposed framework should be read as an interpretive model rather than a comprehensive account of all postpartum return-to-home experiences. No formal critical appraisal of the quality of the sources was undertaken, consistent with the narrative review design; together with the small number of sources and the predominance of correlational, qualitative and non-empirical designs, this means that the findings cannot support conclusions about effectiveness. Discharge readiness was measured at a single point before discharge, and only one study followed mothers after discharge, which limits conclusions about sustained adaptation. Finally, the evidence predominantly reflects mothers’ perspectives: fathers, partners, and professionals were represented in only two sources^26,27^, which limits understanding of the transition from the perspective of the whole family. Future research should test the concept of nested transitions across other clinical contexts, examine its utility for intervention design, and pursue longitudinal studies tracking discharge readiness, self-efficacy, and coping across the early postpartum weeks, alongside subgroup analyses of first-time parents, cesarean recovery, and families with limited socioeconomic resources.

## CONCLUSION

The postpartum return-to-home transition emerged as a multidimensional process shaped by interacting personal, interpersonal and system-level conditions. Parity, mode of birth, infant feeding method, and parental knowledge and confidence were the conditions most frequently identified, while community and health-system factors, particularly the quality, timing, and content of discharge preparation, appeared to influence whether these needs were adequately supported. Discharge readiness was the most frequently measured pattern of response but, as a point-in-time process indicator, it does not demonstrate sustained adaptation, and outcome indicators such as mastery were rarely assessed. Discharge education was the most frequently examined nursing-midwifery therapeutic; the quality of in-hospital teaching was associated with readiness in two correlational studies.

The proposed framework may help inform efforts by nurse-midwives to strengthen discharge preparation, ensure continuity of care, and develop structured referral pathways between hospital and community services. As an interpretive model derived from a small body of evidence, it requires empirical validation, particularly in routine postpartum care.

## Supplementary Material



## Data Availability

Data sharing is not applicable to this article as no new data were created.

## AΙ DECLARATION

During the preparation of this work, the authors used Claude (Anthropic) in order to improve the overall readability of the text. After using this tool/service, the authors thoroughly reviewed and edited the content as needed and take full responsibility for the content of the published article.
