# Supplementary material for: The postpartum return-to-home transition: A narrative review
Source: Eur J Midwifery. 2026 Oct 31;10:10.18332/ejm/241927. doi: 10.18332/ejm/241927 (PMC13741889; doi:10.18332/ejm/241927)
Supplement: Supplementary file 1 [file EJM-10-51-s1.pdf]

EUROPEAN JOURNAL OF MIDWIFERY

Supplementary file

© 2026 Silva, M. A. P. et al.

DOI:
10.18332/ejm/241927

The content has been provided by the author(s) and has not been reviewed, verified, or endorsed by European Publishing. It may not have undergone peer review. The views, opinions, and recommendations expressed are solely those of the author(s) and do not necessarily reflect the position of European Publishing. European Publishing accepts no responsibility or liability for any consequences arising from the use of, or reliance on, this content.

Supplementary Figure S1: PRISMA 2020 flow diagram

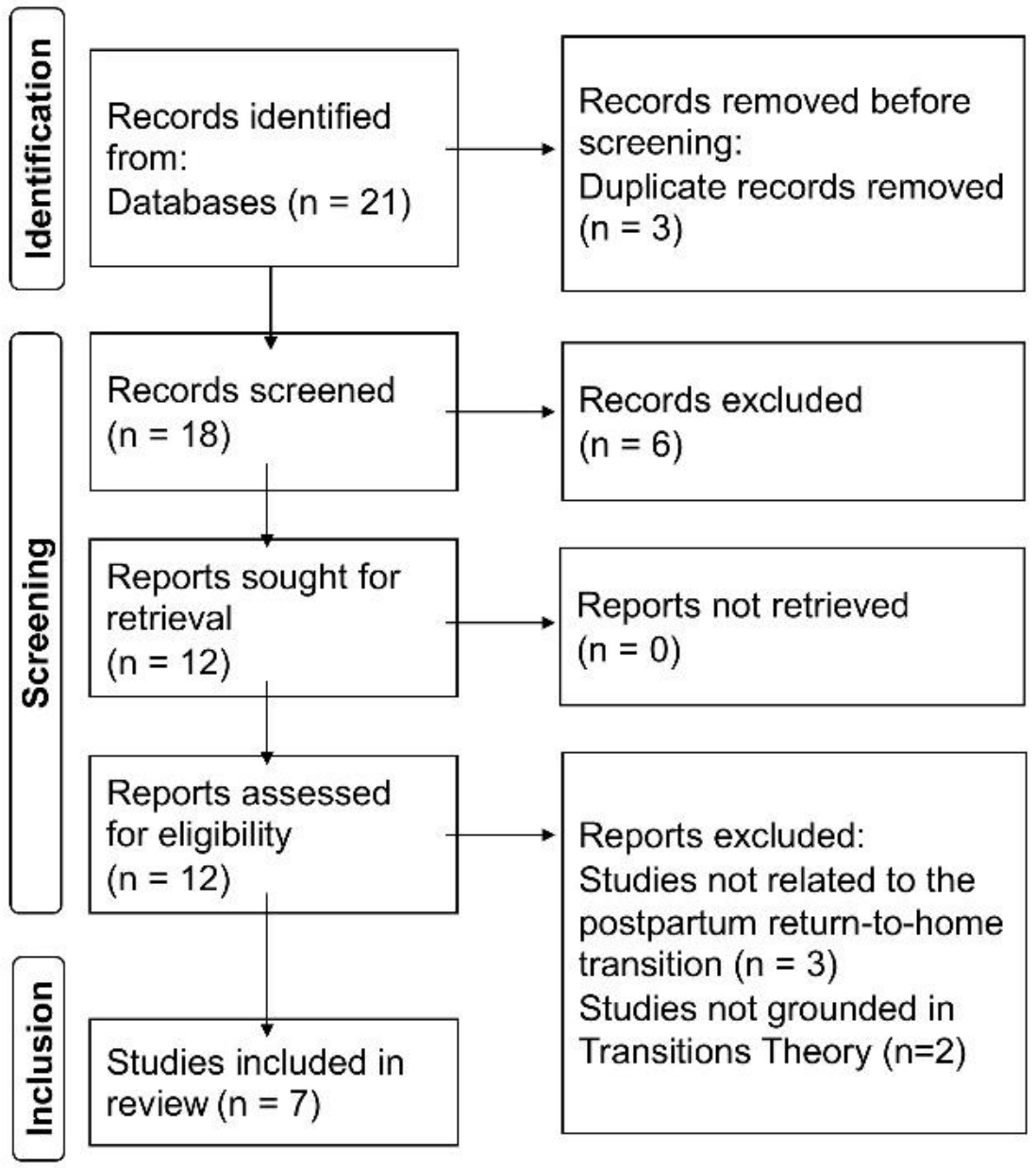

Supplementary Table S1. Characteristics of the seven sources included in the narrative review (PubMed, Scopus and CINAHL, searched on 30 March 2026; n = 7): author, year, country, design, setting and population, use of Transitions Theory and main findings by component of the theory

Author (Year)	Country / Setting	Aim	Design / Sample	Main findings
Bilgiç & Bozkurt (2026)	Turkey – public hospital	To examine the effects of online infant care training given to pregnant women, based on Meleis's Transition Theory, on breastfeeding success and readiness for hospital discharge in the postpartum period.	Randomized controlled trial (n=102)	Transitions Theory used as explicit theoretical and intervention design framework. Multiplicity of transitions acknowledged in framing but not formally theorized. Focus on antenatal period and discharge point. Transition conditions: primiparity (by design), gestational age, mode of birth, education, employment status, income, planned pregnancy, infant sex (all assessed but no significant group differences). Early postpartum discharge policy identified as key system condition. Absence of structured antenatal education described as system-level inhibiting condition. Process indicators: breastfeeding success and discharge readiness.<br>Nursing-midwifery therapeutics: 2 structured antenatal online group sessions: Session 1 - breastfeeding (technique, milk expression, supply signs, infant monitoring, communication); Session 2 - infant care skills (bathing, massage, sleep hygiene, safety, common problems).

Malagon-Maldonado et al. (2017)	USA – postpartum unit in a women's hospital	To examine predictors of readiness for hospital discharge among postpartum women.	Quantitative predictive study involving postpartum mothers following uncomplicated births (n=185).	A mother's perceived readiness for hospital discharge may be influenced by these transition conditions: parity (mothers with three or more children), type of birth (vaginal birth), infant feeding (exclusive bottle-feeding). Nursing-midwifery therapeutics: the quality of discharge teaching (how nurses taught and the content needed) had the greatest influence on discharge readiness.
Weiss & Lokken (2009)	USA – tertiary perinatal centre	To identify predictors and outcomes of postpartum mothers' perceptions of readiness for hospital discharge following childbirth.	Correlational quantitative study involving postpartum mothers (n=141)	Return-to-home transition as a structured, three-phase process (hospitalization – discharge – post discharge) characterized by vulnerability. Process indicators: readiness for discharge and coping difficulty. Outcome indicator: utilization of health services after discharge (calls to provider, office visits, emergency care). Nursing-midwifery therapeutics: Quality of discharge teaching (content needed vs. received and the skills of nurses) improves postpartum mothers' perceptions of discharge readiness.
Hua et al. (2021)	China – tertiary hospital NICU	To explore facilitators and inhibitors of discharge preparation for parents of preterm infants using Transitions Theory.	Qualitative descriptive study using semi-structured interviews with 17 parents and 13 healthcare professionals.	Return-to-home transition conceptualized as a multidimensional process involving simultaneous developmental, situational, and health transitions. Transition conditions include mixed emotions, socioeconomic factors (education, health literacy), and knowledge of infant care, alongside community/system factors such as access to healthcare provider, primary care resources, family support, and reliable internet sources. Process indicator: confidence in caring. Outcome indicator: discharge readiness. Nursing therapeutics: tailored discharge education, early initiation of discharge preparation, and psychological support.

Knudsen et al. (2019)	Denmark – postnatal ward and community health services	To explore first-time parents' experiences of the transition from hospital postnatal care to community health care.	Ethnographic study involving observation and interviews with three first-time parents/couples.	Return-to-home transition described as a multidimensional and vulnerable period involving major life changes and cross-sector transfer of care, where key conditions include parity (first-time parents need more support), alongside system challenges such as early discharge and quality of discharge preparation. Process indicators: parental sense of security and confidence. Nursing-midwifery therapeutics: guidance and information through supportive interactions, transfer of information between hospital and primary care, early home visits.
Boykova (2012)	USA	To explore challenges experienced by parents during the transition from hospital to home following neonatal hospitalization.	Literature review	A complex, multidimensional, nonlinear process involving dual transitions. Social support from partners, extended family and community networks is a facilitating condition. Process indicators: parental confidence/self-efficacy and stress/anxiety/difficulty coping. Outcome indicator: infant rehospitalization.
Orr et al. (2020)	Canada	To apply Meleis' Transitions Theory to understand the NICU-to-home transition experienced by adolescent mothers	Theoretical/ conceptual paper applying Transitions Theory to a specific population	Multiple, intersecting transition, involving situational (NICU-to-home), health-illness (ill infant), and two developmental transitions (becoming an adult and motherhood). Process indicators: stress and uncertainty. The transition experience cannot be understood as a single event and requires a multidimensional, theory-informed approach to support adaptation.
